# Hydrocarbon bio-jet fuel from bioconversion of poplar biomass: life cycle assessment

**DOI:** 10.1186/s13068-016-0582-2

**Published:** 2016-08-11

**Authors:** Erik Budsberg, Jordan T. Crawford, Hannah Morgan, Wei Shan Chin, Renata Bura, Rick Gustafson

**Affiliations:** School of Environmental and Forest Sciences, University of Washington, Box 352100, Seattle, WA 98195-2100 USA

**Keywords:** Acetogen, Bioconversion, Biofuel, Biorefinery, Bio-jet fuel, Life cycle assessment

## Abstract

**Background:**

Bio-jet fuels compatible with current aviation infrastructure are needed as an alternative to petroleum-based jet fuel to lower greenhouse gas emissions and reduce dependence on fossil fuels. Cradle to grave life cycle analysis is used to investigate the global warming potential and fossil fuel use of converting poplar biomass to drop-in bio-jet fuel via a novel bioconversion platform. Unique to the biorefinery designs in this research is an acetogen fermentation step. Following dilute acid pretreatment and enzymatic hydrolysis, poplar biomass is fermented to acetic acid and then distilled, hydroprocessed, and oligomerized to jet fuel. Natural gas steam reforming and lignin gasification are proposed to meet hydrogen demands at the biorefineries. Separate well to wake simulations are performed using the hydrogen production processes to obtain life cycle data. Both biorefinery designs are assessed using natural gas and hog fuel to meet excess heat demands.

**Results:**

Global warming potential of the natural gas steam reforming and lignin gasification bio-jet fuel scenarios range from CO_2_ equivalences of 60 to 66 and 32 to 73 g MJ^−1^, respectively. Fossil fuel usage of the natural gas steam reforming and lignin gasification bio-jet fuel scenarios range from 0.78 to 0.84 and 0.71 to 1.0 MJ MJ^−1^, respectively. Lower values for each impact category result from using hog fuel to meet excess heat/steam demands. Higher values result from using natural gas to meet the excess heat demands.

**Conclusion:**

Bio-jet fuels produced from the bioconversion of poplar biomass reduce the global warming potential and fossil fuel use compared with petroleum-based jet fuel. Production of hydrogen is identified as a major source of greenhouse gas emissions and fossil fuel use in both the natural gas steam reforming and lignin gasification bio-jet simulations. Using hog fuel instead of natural gas to meet heat demands can help lower the global warming potential and fossil fuel use at the biorefineries.

## Background

Political instability in oil-rich regions and the threat of climate change are driving a demand for sustainable biomass-based transportation fuels. Until recently, a significant amount of research in biofuel production has focused on ethanol. A general consensus from this research indicates that ethanol has the potential to reduce greenhouse gas emissions compared with petroleum-based fuels [1, 2]. However, its use is limited by a lack of compatibility with much of the existing transportation infrastructure. This lack of compatibility extends to the aviation sector where the chemical and physical properties of ethanol prohibit its use as an alternative to petroleum-based jet fuel. Attempting to restructure the world’s airline fleet to operate on new type of fuel could cost close to a trillion U.S. dollars [3].

Safety concerns regarding fuel performance and large financial costs associated with a new fuel type dictate that alternative aviation fuels must be chemically identical to the fossil fuels that they intend to replace. Hydrocarbon biofuels, also known as “drop-in” biofuels, meet these requirements. Currently, bio-jet fuel researchers propose to make these fuels from the hydroprocessing of oil seeds, pyrolysis, gasification/Fischer–Tropsch, or advanced fermentation of biomass [4–8]. Like ethanol, these drop-in bio-jet fuels have the potential to significantly reduce GHG emissions. Global warming potential (GWP) for oil seed-based jet fuels is 41–70 % lower than petroleum-based jet (petro-jet) fuel [4, 6, 7]. Bio-jet fuel from pyrolysis of corn stover has GWP reductions of 55–68 % compared with petro-jet [6, 7]. Fischer–Tropsch bio-jet fuel from biomass can reduce the GWP by as much as 81–89 % compared with petro-jet if CO_2_ is sequestered at the biorefinery using a carbon capture system [4, 7]. Using economic factors, Agusdinata et al. [5] project a median GWP reduction of 74 %, compared with petro-jet, for hydroprocessed and Fischer–Tropsch bio-jet fuels in 2050. Staples et al. [8] predict GWP reduction ranges of 130–0.2 % of petro-jet fuel for the advanced fermentation of biomass. This large range is a result of varied feedstock type and conversion methods [8].

The above results present large reductions in the GWP compared with the baseline petroleum jet fuel; however, none of these theoretical GWP values of bio-jet fuel include land-use change emissions. Land-use change (both direct and indirect) emissions have been left out of many biofuel life cycle assessment (LCA) studies as there is still a great deal of uncertainty in land-use change models, especially for oil seed feedstocks [7]. If land-use change emissions for oil seeds are applied to the oil seed LCAs, the GWP of the biofuels increases significantly [7]. Stratton et al. [4] assessed different simulations, included land-use change emissions in some of these, and found that when oil seed simulations include land-use change the GWP is estimated to increase from 22 % to as much 2000 %. Adding direct land-use change emissions to bio-jet fuels fermented from switchgrass could increase the GWP CO_2_ eq. values by 2.9–12.2 g MJ^−1^ [8].

To date limited research has been reported on drop-in biofuels that use the bioconversion platform that has been widely modeled to produce ethanol [8]. A benefit of this platform is the ability to use a wide range of feedstocks such as corn, sugarcane, switchgrass, corn stover, and short rotation woody crops (SRWC) [1, 2, 8]; many of which—such as switchgrass and SWRC—can be cultivated on marginal lands [9, 10]. Cultivating marginal lands for growing biomass for biofuels can potentially help limit direct and indirect land-use change emissions [2]. The advanced fermentation work of Staples et al. [8] considers using sugar cane, corn grain, and switchgrass along with pretreatment and bioconversion techniques appropriate for each feedstock. The survey research is theoretically based and calculates yields and emissions that identify life cycle GWP ranges from producing and using middle distillates (diesel and jet fuel). As noted above, the authors predict significant GWP reductions, but that the results vary and are dependent on the feedstock and conversion methods used [8]. The large range in GWP values reported in Staples et al. [8], and other biofuel LCAs [1, 4–7], indicate the sensitivity of the GWP to feedstock and conversion method, necessitating the need for a detailed LCA of any biofuel production method proposed for commercial scale application.

The work presented here is part of an investigation into the environmental and economic impacts of bio-jet fuel commercially produced via an advanced bioconversion pathway. The research focuses on the use of a novel fermentation method to convert poplar biomass to jet fuel. The work is part of a collaboration between industry, government, and academia to develop a sustainable biofuels and biochemicals industry. In this article, LCA is used to determine potential environmental impacts. A second article, also published in this journal, investigates the techno-economics of the proposed bio-jet fuel pathway and includes detailed descriptions of bioconversion processes [11].

Poplar biomass, a SRWC, is selected as the feedstock as it presents an attractive option for diversifying and expanding biomass available for biofuel production. Used in the past for various products such as fuel wood, lumber, and paper, these well-established crops present good characteristics for biofuel use. In general, they require little fertilizer input, have the ability to resprout after multiple harvests, and have a high biomass production [9]. The lignocellulosic material in the wood can also be fractionated without extensive pretreatment [12], and hardwoods do not exhibit the recalcitrance reported in softwoods [13]. Research has shown that these trees can be grown on marginal lands [9]. Using marginal/fallow lands is unlikely to induce large land-use change emissions [2], potentially avoiding the land use concerns observed with oil seeds and food crops.

An acetogen fermentation pathway is used in the biorefinery to convert lignocellulosic material to liquid fuel. The acetogen pathway is chosen over an ethanologen pathway because of its ability to achieve a much higher fuel yield. During digestion of sugars, ethanologens produce 1 mol of CO_2_ for every mol of ethanol produced. This limits the theoretical ethanol yield at 67 % [14]. Acetogens only produce acetic acid as they digest sugars and have a theoretical yield of 100 % [15]. Fermentation of sugars from biomass to acetic acid using *Moorella thermoacetica* is being investigated in our laboratories at the University of Washington [16]. Yields and titers depend on glucose to xylose ratios and the presence of compounds such as phenolics, furfural, and hydroxymethylfurfural. It has been found that conversion yields of over 70 % and titers of 40 g L^−1^ are readily achievable. Higher yields and titers have been reported by Crawford et al. [11] based on data from our industry partners. The acetic acid is then converted to ethanol [17]. No carbon is lost as CO_2_ and the ethanol yield is 80 % higher than if an ethanologen were used [18]. From here, the ethanol is then upgraded to jet fuel via dehydration, oligomerization, and hydroprocessing [17].

Jet fuel is a complex mixture of a large number of different hydrocarbons. The focus of this research is on the life cycle assessment of producing biomass-based molecules that could be readily incorporated into the jet fuel supply chain. *n*-Dodecane, C_12_H_26_, is a good first approximation for kerosene-type jet fuel and has an appropriate carbon number of 12 [19]. The carbon number is important for calculating the degree of oligomerization required. Dodecane also has similar density, viscosity, thermal conductivity, and heat capacity to kerosene-based jet fuel [20]. It is therefore chosen as the end product (i.e., bio-jet fuel) to be modeled in this work.

It should be noted that the acetogen-based biorefinery could also be used to produce acetic acid, ethanol, ethylene, and ethyl acetate. All of these chemicals are intermediates produced—some at high purities—in route to bio-jet fuel production. Details of these other chemicals that can be produced in the modeled process are discussed in Crawford et al. [11].

Cradle to grave (well to wake) LCAs are conducted to investigate the life cycle impacts of four production scenarios for the conversion of poplar tree chips into jet fuel via fermentation and subsequent hydrogenation. The goal of producing bio-jet fuel from poplar biomass is to create an alternative to petroleum-based jet fuel (petro-jet). In addition to assessing the overall life cycle impacts of producing bio-jet fuel from poplar biomass via a bioconversion route, different methods for obtaining hydrogen and meeting heat and steam demands are investigated. Hydrogenation steps are a crucial part of upgrading acetic acid to a hydrocarbon biofuel. Two hydrogen production methods, natural gas steam reforming (NGSR) and lignin gasification (LG), are integrated into biorefinery system simulations to determine the effect of each process in producing hydrogen. Biorefinery heat and steam demands cannot be entirely met from combustion of lignin, as is commonplace in many second-generation ethanol biorefineries [1, 18], and energy must be imported. Natural gas and hog fuel are tested as energy sources to meet these energy demands.

The hog fuel used in the simulations is assumed to be an unprocessed mix of woody biomass (chips, bark, residue, etc.) produced as a by-product of lumber mill operations. Burning hog fuel allows for the use of a renewable energy source in place of fossil fuel. CO_2_ released from burning the hog fuel is of biogenic origin and will not contribute to the net GWP of the jet fuel as compared to burning natural gas which releases nonbiogenic CO_2_ and increases the net global warming potential of the jet fuel. However, using hog fuel is not completely free of nonbiogenic greenhouse gas emissions as GHGs are emitted during the production and transportation of hog fuel.

In total, four biorefinery designs are assessed to make bio-jet fuel: NGSR used to produce hydrogen with natural gas and lignin burned for heat and steam (NGSR bio-jet), NGSR for hydrogen with hog fuel and lignin burned for heat and steam (NGSR-HF bio-jet), LG used for hydrogen production and natural gas for heat and steam (LG bio-jet), and LG for hydrogen and hog fuel for heat and steam (LG-HF bio-jet). One mega joule (MJ) of jet fuel combusted in a jet engine is used as the functional unit. The bio-jet fuel results are compared to each other, as well as petro-jet.

## Results

The following categories are used to identify the areas within the system boundaries that contribute to the environmental impacts.

- *Carbon in biomass* CO_2_ absorbed by photosynthesis in harvested chips, above ground and below ground stumps, and coarse roots modeled over 21 years. The equivalent amount of CO_2_ stored in the poplar wood is calculated using the stoichiometric relationship of CO_2_ to carbon of 3.66 kg kg^−1^.
- *Poplar growth and harvesting* All technosphere processes associated with growing and harvesting poplar; includes direct land-use change emissions.
- *Ancillary chemicals* All chemical inputs to the biorefinery.
- *Transportation* Includes transportation of poplar from farm to biorefinery gate, all chemical inputs to the biorefinery, and bio-jet fuel from the biorefinery to a distribution center.
- *Biorefinery* All operations performed, raw materials used, and emissions from each biorefinery.
- *Avoided production (NGSR-based bio-jet fuels)* Avoided production of electricity resulting from the production of excess electricity. It is assumed that marginal electricity, created by natural gas combustion, will be displaced.
- *Purchased electricity (LG-based bio-jet fuels)* Electricity needed for biorefinery operations.
- *Jet fuel use* Combustion of jet fuel in a jet engine.
- *Petro-jet fuel production and use* Life cycle data for the production and use of petroleum-based jet fuel are obtained from the greenhouse gases, regulated emissions, and energy use in transportation model v1.2.0.11425 (GREET).

### Global warming potential

**Fig. 1:**
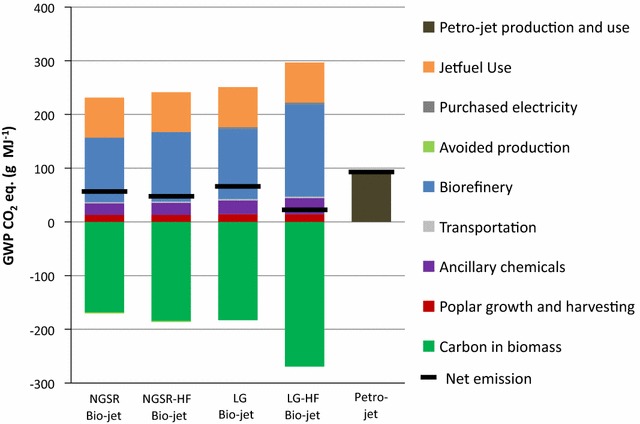
Cradle to grave global warming potentials of bio-jet fuel production simulations. Petro-jet shown for comparison

GWP values, as well as contributions from each area within the biorefinery life cycles, are shown in Fig. 1 for the four bio-jet simulations and petro-jet. Net GWP for each simulation are listed in Table 1. For all simulations, the biorefinery category is the largest source of greenhouse gases (GHGs) that contribute to the GWP and jet fuel use is the second largest. However, all of the CO_2_ emissions from jet fuel use and most of the CO_2_ emissions from the biorefineries are from biogenic sources and are offset by the CO_2_ that was removed from the atmosphere and stored as carbon in the biomass during growth. When biogenic CO_2_ emissions are removed from the GWP calculation (by subtracting biogenic CO_2_ in from biogenic CO_2_ out), the biorefinery is still the largest source of CO_2_ for the NGSR, NGSR-HF, and LG bio-jet simulations, but the ancillary chemicals become the second largest contributor to the GWPs. In the LG-HF bio-jet process, the ancillary chemicals group has the largest impact on the GWP when biogenic CO_2_ emissions are removed. The largest contributions to the GWP of poplar growth and harvesting are direct land-use change and nitrogen fertilizer. Direct land-use change emissions contribute GWP CO_2_ eq. of 12 g MJ^−1^ of bio-jet fuel to each simulation. Processes associated with manufacturing nitrogen fertilizer and N_2_O emissions resulting from its use generate GWP CO_2_ eq. of 5 g MJ^−1^ for each bio-jet fuel simulation. Transportation and purchased electricity (LG and LG-HF bio-jet) comprise only small percentages of the GWPs. Avoided production credit for NGSR and NGSR-HF bio-jet has little effect in reducing the net GWP of the two bio-jet processes. Biorefinery and ancillary chemical GWP contributions are discussed in more detail below.

**Table 1:** Global warming potentials and fossil fuel use for four bio-jet production simulations

Impact	NGSR bio-jet	NGSR-HF bio-jet	LG bio-jet	LG-HF bio-jet	Petro-jet
Net GWP CO_2_ eq. (g MJ^−1^)	66	60	73	32	93
Net fossil fuel use (MJ MJ^−1^)	0.84	0.78	1.0	0.71	1.2

**Fig. 2:**
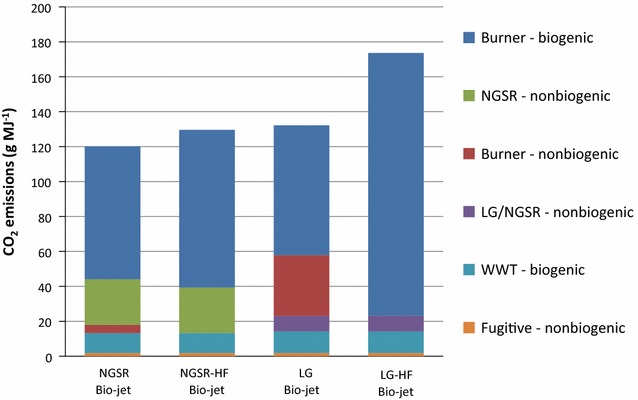
Biorefinery CO_2_ emission sources for each biorefinery configuration. Emissions are identified as being from either a biogenic or nonbiogenic source. NGSR refers to emissions from natural gas steam reforming. LG/NGSR refers to emissions from NGSR to produce supplemental hydrogen in the lignin gasification simulations. WWT refers to emissions from the onsite wastewater treatment facility

GHGs emitted from the biorefineries are almost entirely CO_2_. Sources of the CO_2_ emissions for each biorefinery are shown in Fig. 2. CO_2_ emissions in Fig. 2 are identified as biogenic or nonbiogenic. In all four simulations, the largest source of biogenic CO_2_ is the burning of biomass. In the NGSR bio-jet, this biomass is the lignin in the burner/boiler. For NGSR-HF, this is lignin and hog fuel in the burner/boiler. NGSR-HF bio-jet emits more CO_2_ than NGSR bio-jet, but the increase is from a biogenic source and do not increase the net GWP. The result is a lower net GWP for NGSR-HF compared with NGSR bio-jet (Table 1).

In the LG bio-jet, the lignin gasification process emits CO_2_ of biogenic source. Natural gas steam reforming to produce the supplemental hydrogen emits nonbiogenic CO_2_ (LG/NGSR—nonbiogenic, Fig. 2). To meet the heat and steam demand in the LG bio-jet simulation, significantly more natural gas for heat and steam must be used than in NGSR bio-jet. Combustion of this natural gas is the largest source of nonbiogenic CO_2_ emissions in LG bio-jet (Burner—nonbiogenic, Fig. 2). In the LG-HF biorefinery, burning hog fuel increases biogenic CO_2_ emissions and decreases nonbiogenic CO_2_ emissions, achieving a net reduction in the GWP compared with LG bio-jet (Table 1). Wastewater treatment and fugitive CO_2_ emissions are minor and the same for all four simulations.

**Fig. 3:**
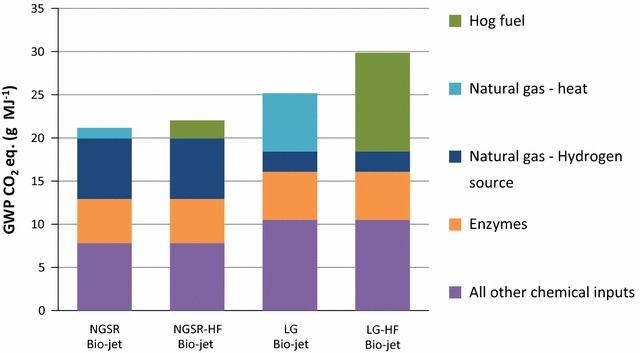
Global warming potential: ancillary chemicals. The top four global warming potential contributors are identified for each biorefinery simulation. All other chemical inputs are grouped together

In the ancillary chemicals category, the processes that contribute the most to the GWP depends on the bio-jet fuel production method (Fig. 3). For NGSR and NGSR-HF bio-jet, these are the manufacturing of enzymes and the acquisition/production of natural gas to be used in hydrogen production. These two processes produce GWP CO_2_ eq. of 7.0 and 5.1 g MJ^−1^ jet fuel, respectively. Acquisition/production of natural gas for heating only contribute GWP CO_2_ eq. of 1.2 g MJ^−1^ to the NGSR bio-jet GWP. Acquiring hog fuel to produce heat and steam in NGSR-HF bio-jet produces GWP CO_2_ eq. of 2.1 g MJ^−1^.

The largest contributors from the ancillary chemicals in the LG bio-jet GWP are the acquisition/production of natural gas for heat and steam, and the production of enzymes. These two processes produce GWP CO_2_ eq. of 6.7 and 5.5 g MJ^−1^, respectively. Natural gas for supplemental hydrogen production creates GWP CO_2_ eq. of 2.4 g MJ^−1^. In the LG-HF bio-jet process, acquisition of hog fuel for heat and steam produces GWP CO_2_ eq. of 11 g MJ^−1^. Production of enzymes and acquisition/production of natural gas for supplemental hydrogen production contribute GWP CO_2_ eq. of 5.5 and 2.4 g MJ^−1^, respectively, to the LG-HF bio-jet GWP (Fig. 3).

### Fossil fuel use

**Fig. 4:**
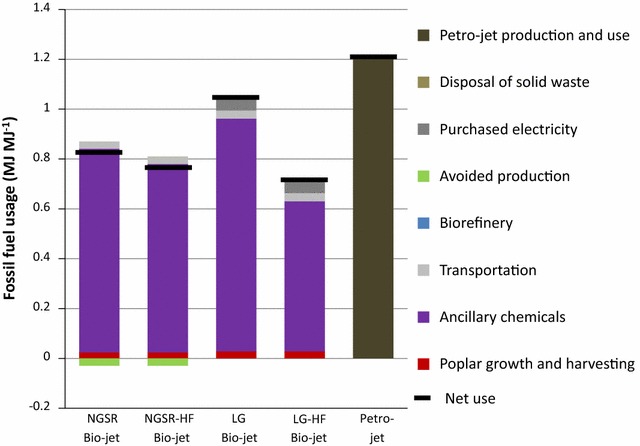
Fossil fuel use for each biorefinery process. Petro-jet shown for comparison

FFU values of each biorefinery are presented in Fig. 4. Net FFU values for each simulation are listed in Table 1. Acquisition/manufacturing of products within the ancillary chemicals use 85–97 % of the fossil fuels consumed in the making of the bio-jet fuels. FFU in transportation, poplar growth and harvesting, and purchased electricity (for LG and LG-HF bio-jet) are minor compared with the ancillary chemicals group (Fig. 4). Avoided production credit in NGSR and NGSR-HF bio-jet simulations reduces the net FFU by 0.029 MJ MJ^−1^ of jet fuel.

**Fig. 5:**
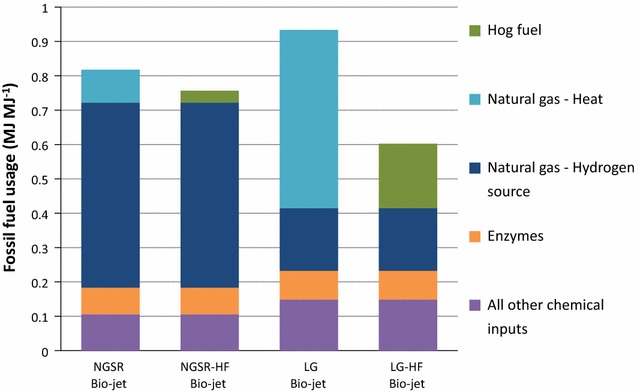
Fossil fuel use: ancillary chemicals. The top four fossil fuel consumers are identified for each biorefinery simulation. All other chemical inputs are grouped together

The largest consumers of fossil fuels in the ancillary chemicals group depend on the bio-jet method (Fig. 5). In the NGSR and NGSR-HF simulations, enzyme production and natural gas use for hydrogen production are the largest users of fossil fuels. Acquisition/production of natural gas for hydrogen production and enzyme production use 0.54 and 0.077 MJ MJ^−1^, respectively. Natural gas for heating consumes 0.096 MJ MJ^−1^ and is the third largest consumer of fossil fuels in NGSR bio-jet. Acquiring hog fuel requires 0.035 MJ MJ^−1^ in NGSR-HF bio-jet. All other ancillary chemicals in NGSR and NGSR-HF bio-jet combine to use 0.10 MJ MJ^−1^.

In LG, bio-jet natural gas for heat and steam is the largest consumer of fossil fuels (0.52 MJ fossil fuel MJ^−1^). In LG-HF, bio-jet hog fuel acquisition is the largest consumer of fossil fuels, requiring 0.19 MJ MJ^−1^. In both the LG and LG-HF bio-jet simulations natural gas for supplemental hydrogen production is the second largest user of fossil fuels, at 0.18 MJ MJ^−1^, followed by enzyme production which uses 0.084 MJ MJ^−1^. All other ancillary chemicals in the LG and LG-HF bio-jet pathways combine to use 0.15 MJ MJ^−1^.

## Discussion

All four bio-jet pathways have lower GWP and FFU values compared with petroleum-based jet fuel. The range of the GWP and FFU values for the bio-jet fuels are based on the method chosen to produce hydrogen and the energy source used to provide heat and steam at the biorefineries (Table 1). NGSR-HF bio-jet pathway has a net GWP that is 9 % lower than NGSR bio-jet. Net FFU is reduced by 8 % using hog fuel to replace natural gas for steam production. The similarity between the two NGSR-based bio-jet pathways is expected as the only difference between the two pathways is the energy source that provides 14 % of the total heat and steam demand. This is in contrast to the LG bio-jet pathways, which require that all heat and steam demand is met by an external source of energy. These processes are more sensitive to the type of external energy source used. Net GWP of LG-HF bio-jet is 56 % lower than LG bio-jet pathway. FFU is 46 % lower in the LG-HF compared with the LG process.

Replacing natural gas with hog fuel reduces the GHGs released and fossil fuels used during the acquisition, production, and burning of natural gas. Using hog fuel does not completely eliminate all GHG emissions and fossil fuel use associated with heat and steam production. GHGs are released and fossil fuel fuels are consumed during the production and transportation of hog fuel. Replacing 1 MJ of natural gas with 1 MJ of hog fuel reduces the GWP CO_2_ eq. by 60 g and fossil fuel use by 0.65 MJ. These results indicate that from a life cycle impact standpoint, hog fuel is the preferred energy source for heat and steam generation in the biorefineries.

The hydrogen production method used is responsible for driving much of the difference in the bio-jet fuels life cycle GWPs and FFUs. The hydrogen process selected determines the amount of excess energy needed to meet the heat and steam demand of the biorefinery. If natural gas is used to meet the energy demand, the NGSR bio-jet process has a lower net GWP and FFU than LG bio-jet. NGSR bio-jet would be the preferred option. If hog fuel is used to meet the energy demand, LG-HF bio-jet simulation is the best option, not only between the two hog fuel simulations, but also of all four simulations assessed. LG-HF bio-jet has significantly lower GWP and FFU values than the other three bio-jet simulations (Table 1). Techno-economic assessment of these four bio-jet simulations, however, finds that gasifying lignin is more expensive and complicated than natural gas steam reforming. As technology improves gasifying lignin may become financially feasible, but at this time, the cheapest pathway to bio-jet fuel production with the lowest lifecycle impacts is using natural gas to produce hydrogen and hog fuel to meet the excess heat/steam demand [11].

The reductions of GWPs compared to petro-jet reported in this study are not as large as those reported in the literature (except for LG-HF) [4–8]. The system boundaries in this study include direct land-use change emissions and are partly responsible for the higher GWP values. If direct land-use change emissions are removed from the system boundaries of this study, the GWP reductions for NGSR, NGSR-HF, LG, and LG-HF bio-jet pathways become 42, 49, 34, and 78 %, respectively, compared with petro-jet. These reductions are similar to the lower range of reductions reported for hydroprocessed seed oil (41–74 %) [4, 6, 7], pyrolysis bio-jet fuels (55–68 %) [6, 7], and advanced fermentation of switchgrass to middle distillates (0.2–78 %) [8]. LG-HF (without land-use change) achieves a GWP similar to the Fischer–Tropsch bio-jet fuels that include carbon capture systems (74–89 %) [4, 5, 7]. The incorporation of land-use change models into bio-jet LCAs is important to understand the true impact these fuels could have on climate change.

Process designs specific to the bioconversion platform also drive differences in the GWP values of the biorefinery configurations simulated in this study versus those in the literature. The fermentation of switchgrass to ethanol and subsequent dehydration, oligomerization, and hydroprocessing in Staples et al. [8] is most similar to the biorefinery design used in this study. In that report, the GWP CO_2_ eq. of jet fuel produced from switchgrass using an ethanol fermentation platform is found to be 11.7 g MJ^−1^ and increases by 2.9–12.2 g MJ^−1^ if direct land-use change emissions are included. This is lower than the GWP of NGSR, NGSR-HF, LG, and LG-HF simulations (Table 1). The choice of fermentation pathway is largely responsible for the difference in GWP values. Fermenting to acetic acid and then converting it to ethanol achieve a much higher yield compared with fermenting straight to ethanol; however, hydrogen is required to convert the acetic acid to ethanol. 93 % of all hydrogen required in the NGSR and LG biorefinery simulations is needed to convert acetic acid to ethanol. Avoiding this step in the conversion of biomass to jet fuel would reduce GWP, but tradeoffs between final product yield and selling price need to be accounted for as well. A higher biofuel yield also reduces the amount of land needed. It is estimated that there is enough marginal/abandoned land to meet 26–55 % of the current world liquid fuel demand using second-generation biofuel production methods [21]. The acetogen pathway is approximated to produce 80 % more ethanol than the ethanologen pathway and could increase the amount of liquid fuel produced from these agriculturally degraded lands [18]. This equates to a higher jet fuel yield per unit of land that is not possible from a traditional ethanologen fermentation pathway.

A commonality among bio-jet fuels from hydroprocessing of seed oils, pyrolysis, and bioconversion are the hydrogenation step(s) needed to produce the final product. Producing hydrogen is found to be a significant source of GHGs [4, 7] and increased cost [22]. In previous studies, it is assumed that this hydrogen would come from natural gas steam reforming [4, 7]. In this research, lignin gasification to produce hydrogen is shown to have the potential to significantly reduce the GWP of hydrogen production if hog fuel is used to meet the heat and steam demand of the biorefinery. Future studies should assess the integration of other hydrogen production methods in biorefinery designs to determine their economic and environmental viability.

## Conclusion

Bio-jet fuels produced from the bioconversion of poplar biomass reduce the GWP and FFU compared with petroleum-based jet fuel. The production and use of hydrogen are identified as major contributors to the GWP and FFU of bio-jet fuel production. The hydrogen production method will dictate GHG emissions, FFU, and the degree to which these impacts can be reduced. LCA work in this study suggests that for biorefineries with integrated hydrogen production facilities, using hog fuel in place of natural gas to provide heat and steam will have reduced nonbiogenic CO_2_ emissions and FFU. Baseline biorefinery designs (NGSR and LG bio-jet) already include the infrastructure necessary for burning biomass, and addition of hog fuel to the burner/boiler would not drastically increase capital costs.

The LCA work presented here provides additional evidence that the GWP and FFU of the aviation sector will be reduced if biomass-based drop-in jet fuels replace petroleum-based jet fuels. However, this research is not complete as it only assesses impacts targeting climate change and use/dependence of fossil fuels. Additional research is needed to assess regional environmental impacts such as air quality and water degradation. Locations of biorefineries and affected watersheds must be identified, however, to accurately address these impacts. Future work will report on these issues.

## Methods

Environmental impacts considered are the 100-year Global Warming Potential (GWP) [23] and fossil fuel use. Fossil fuel use (FFU) is calculated by summing all fossil fuel inputs (coal, natural gas, and crude oil) per MJ of jet fuel. Guidelines for conducting a LCA are set by ISO 14040 and 14044 [24, 25], and this research follows the ISO design. The LCAs in this research were developed using SimaPro v.8.0.

**Fig. 6:**
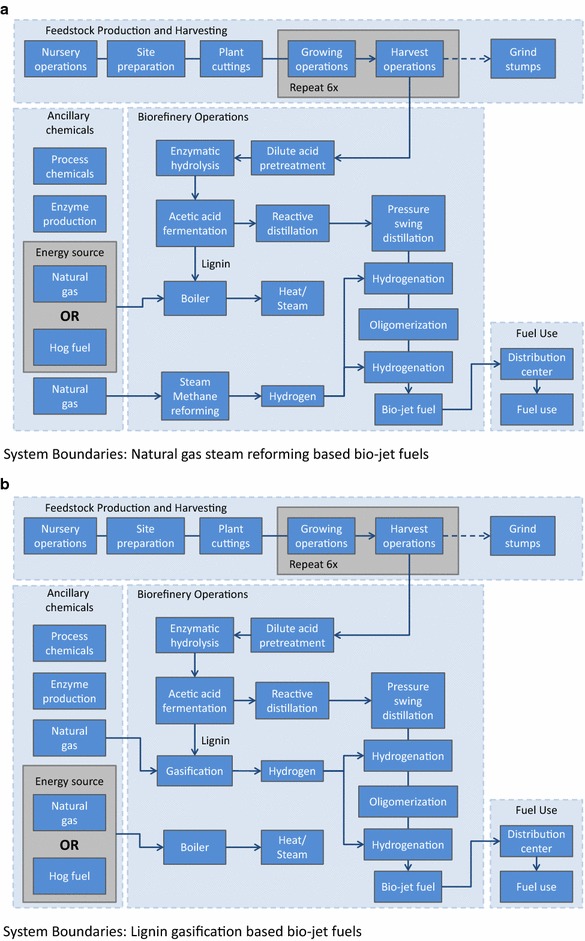
Life cycle inventory system boundaries. **a** Overview of the natural gas steam reforming-based bio-jet fuel process. The type of energy source going to the boiler depends on the biorefinery simulation. NGSR bio-jet uses natural gas and lignin. NGSR-HF uses hog fuel and lignin. Not pictured, but included in the system boundaries are a waste water treatment facility, disposal of solid wastes, and an excess electricity by-product. **b** Overview of the lignin gasification-based bio-jet fuel process. The type of energy source going to the boiler depends on the biorefinery simulation. LG bio-jet uses natural gas. LG-HF uses hog fuel. Not pictured, but included in the system boundaries are a waste water treatment facility, disposal of solid wastes, and import of electricity

The bio-jet fuel life cycles are broken up into 4 sections: feedstock production and harvesting, ancillary chemicals, the biorefinery, and fuel distribution and use. Feedstock production and harvesting and fuel distribution and use are the same for each biorefinery configuration. Biorefinery process and ancillary chemical inputs will vary depending on the configuration. Descriptions of each life cycle section follow below. System boundary diagrams for each bioconversion pathway are displayed in Fig. 6a, b.

### Feedstock

The feedstock production and harvesting model is supported by operational data from industry (GreenWood Resources, personal communication, 2011–2014), literature [26], and LCA databases [27, 28]. It is the same feedstock model used in [18] and is discussed in more detail in that publication. The feedstock production and harvest model is representative of a coppice harvest system, with the poplar trees being coppiced every 3 years for 6 cycles. The model includes all necessary site preparation, nursery operations, management of the poplar tree stands, harvest operations, and stump removal. Nitrogen fertilizer is applied in the spring following a harvest at a rate of 56 kg N per application. N_2_O emissions from fertilizer and decaying biomass are calculated using the Farm Energy Analysis Tool [29]. Storage of carbon in the harvested poplar biomass as well as in below ground biomass (stump and roots) is included. The equivalent amount of CO_2_ stored in the poplar wood is calculated using the stoichiometric relationship of CO_2_ to carbon of 3.66 kg kg^−1^and a carbon mass fraction of 51.7 % dry wood weight [30] (Tables 2, 3). Direct land-use change is included using the assumption that fallow land will be used for poplar plantations. Direct land-use change associated with establishing the plantation is calculated using the Forest Industry Carbon Assessment Tool v.1.3.1.1. Indirect land-use change is excluded from the system boundaries due to uncertainty associated with these models [31]. A transportation distance of 100 km roundtrip is assumed to transport the harvested poplar biomass to the biorefinery gate. In total, the feedstock production and harvest model covers a 21-year timespan [18].

**Table 2:** Chemical mass fraction of bone dry poplar biomass

	Mass fraction of bone dry poplar biomass (%)
Cellulose	42
Xylan	15.3
Lignin	25.8
C5SOLD	1.91
C6SOLD	5.73
Acetate	2.86
Extractives	4.5
Ash	1.91

Xylan, five carbon polysaccharides (C5SOLD) (other than xylan), and six carbon polysaccharides (C6SOLD) combined represent the hemicellulose content [30]

**Table 3:** Elemental composition bone dry poplar biomass

Unit = kg	C	H	O	N	S
Cellulose	0.187	0.0262	0.207	0	0
Xylan	0.0695	0.00936	0.0741	0	0
Lignin	0.200	0.0234	0.0346	0	0
C5SOLD	0.00868	0.00117	0.00925	0	0
C6SOLD	0.0255	0.00357	0.0283	0	0
Acetate	0.0114	0.00192	0.0152	0	0
Extractives	0.00550	5.07E−05	0.0371	0.00235	4.09E−05
Total	0.507	0.0656	0.406	0.00235	4.09E−05
%	51.7	6.69	41.4	0.239	0.00417

Xylan, five carbon polysaccharides (C5SOLD) (other than xylan), and six carbon polysaccharides (C6SOLD) combined represent the hemicellulose content [30]

### Biorefinery

Currently no commercial facilities are producing bio-jet fuel from biomass via a bioconversion process. ASPEN-Plus chemical engineering software is used to simulate potential biorefinery process designs. The biorefinery simulations are designed to operate on 3200 tonnes of bone dry biomass per day. It is assumed that there is no biomass stored on site. Proposed poplar management schemes follow a just-in-time harvest approach wherein poplar is harvested and delivered to the biorefinery as needed, this will avoid emissions associated with long-term biomass storage. Simulation results show poplar biomass to bio-jet fuel conversion yields of 330 L t^−1^ for the natural steam gas reforming-based processes and 305 L t^−1^ for the lignin gasification-based processes. The biorefineries differ from each other in how hydrogen is acquired and the fuel source used to meet the energy demands of the facilities. The descriptions given below are overviews of the processes to convert poplar chips to jet fuel that were investigated. For in-depth descriptions of the biorefinery processes, see the companion techno-economic analysis by Crawford et al. [11].

The biorefineries are similar in their process design and have many of the same unit processes. The processes begin with dilute acid pretreatment and enzymatic hydrolysis. These steps are based on National Renewable Energy Laboratory (NREL) corn stover model, but modified to use a poplar feedstock [31]. Following enzymatic hydrolysis, glucose and xylose are fermented to acetic acid using *Moorella thermoacetica*. Acetic acid fermentation yield is projected to be 90 % and the titer 50 g L^−1^. The yield and titer were supplied by industry partners developing an acetogen-based biorefinery (Tim Eggeman, personal communication, 2014). The acetic acid undergoes reactive distillation, pressure swing distillation, hydrogenation, oligomerization, and one more hydrogenation step to become jet fuel. Biorefinery hydrogen requirement is 122 g L^−1^ of jet fuel. Hydrogen use for the first hydrogenation step, used to convert acetic acid to ethanol, is 114 g L^−1^ of jet fuel. The second hydrogenation step, converting unsaturated hydrocarbons to polymer fuel, uses 8 g L^−1^ jet fuel.

Waste water is treated onsite in a waste water treatment (WWT) plant. The WWT design is based on Humbird et al. [32]. Methane generated during the anaerobic digestion stage is sent to the burner and combusted. All wastes are collected and sent to a landfill for disposal. Descriptions of the hydrogen production processes and energy sources for the four simulations follow below.

#### Simulation 1: Natural gas steam reforming (NGSR bio-jet)

A natural gas steam reforming (NGSR) method is used to provide the hydrogen for the hydrogenation steps. The NGSR facility is integrated into the biorefinery to improve thermal efficiency. In this process, lignin from the poplar biomass is combusted to provide heat and steam for the biorefinery. However, unlike second-generation ethanol bioconversion facilities [32], the combustion of lignin cannot meet the entire heat and steam demand. Combustion of lignin and unreacted carbohydrates produces enough energy to meet 86 % of the heat/steam demand. Natural gas is combusted to make up the remaining 14 % of the demand. Electricity is produced by converting high-pressure steam into electricity via a multistage turbine. A small of amount excess electricity is generated during from process and is sold as a by-product. In the LCA, this by-product is treated using the system expansion method per ISO 14044 guidelines [25]. An avoided production credit is generated for displacing electricity produced from natural gas, a likely candidate for marginal electricity [33]. Major process inputs and outputs for the NGSR bio-jet simulation are listed in Table 4.

**Table 4:** Natural gas steam reforming-based bio-jet processes—major process inputs and outputs referenced to 1 MJ of NGSR and NGSR-HF bio-jet fuels

	NGSR bio-jet	NGSR-HF bio-jet	Unit
Input			
Feedstock (bone dry)	81.4	81.4	g
Enzymes	0.683	0.683	g
Sulfuric acid	1.46	1.46	g
Lime	2.43	2.43	g
Calcium carbonate	0.391	0.391	g
Carbon dioxide	0.172	0.172	g
Ammonia	1.03	1.03	g
Corn steep liquor	2.18	2.18	g
Sodium hydroxide	1.94	1.94	g
Natural gas—steam reforming	0.0525	0.0525	MJ
Natural gas—heat/steam	0.0937	0	MJ
Hog fuel—heat/steam	0	8.23	g
Output			
Bio-jet fuel	1	1	MJ
Excess electricity	0.00254	0.00254	Kwh
CO_2_ (biogenic)	87.5	102	g
CO_2_ (nonbiogenic)	32.6	27.9	g

#### Simulation 2: Natural gas steam reforming with hog fuel (NGSR-HF bio-jet)

This process is similar to the NGSR bio-jet process but attempts to reduce GHG emissions by reducing natural gas use. Natural gas needed to provide heat and steam is replaced with hog fuel. As a result, only biomass (lignin and hog fuel), is used to meet heat and steam demands. Major process inputs and outputs for the NGSR-HF bio-jet simulation are listed in Table 4. When the total amount of biomass used to produce bio-jet fuel is factored into yield calculations (poplar + hog fuel), the NGSR-HF bio-jet fuel yield is 300 L t^−1^.

#### Simulation 3: Lignin gasification (LG bio-jet)

In this process, lignin from the poplar biomass is recovered after fermentation and sent to a gasifier integrated into the biorefinery to produce hydrogen. The lignin gasification can only meet 75 % of the facilities hydrogen demand. Natural gas steam reforming is used to supplement the remaining 25 %. In the LG bio-jet process, all lignin is consumed in the gasification process and cannot be combusted to provide energy for the biorefinery. Natural gas is combusted in the burner/boiler of the biorefinery to provide the necessary heat and steam. High-pressure steam is converted to electricity via a multistage turbine, but this alone cannot meet the entire biorefinery electricity demand. The remaining electricity demand is met by purchasing electricity. For the life cycle inventory work, a unit process representing the average makeup of the 2012 U.S. national electrical grid is used to supply electricity [34]. Major process inputs and outputs for the LG bio-jet simulation are listed in Table 5.

**Table 5:** Lignin gasification-based bio-jet processes—major process inputs and outputs referenced to 1 MJ of LG and LG-HF bio-jet fuels

	LG bio-jet	LG-HF bio-jet	Unit
Input			
Feedstock (bone dry)	88.4	88.4	g
Enzymes	0.743	0.743	g
Sulfuric acid	1.59	1.59	g
Lime	2.64	2.64	g
Calcium carbonate	0.425	0.425	g
Carbon dioxide	0.187	0.187	g
Ammonia	1.12	1.12	g
Corn steep liquor	2.37	2.37	g
Sodium hydroxide	1.36	1.36	g
Natural gas—supplemental hydrogen source	0.178	0.178	MJ
Natural gas—heat/steam	0.506	0	MJ
Hog fuel—heat/steam	0	44.5	g
Electricity	0.253	0.253	kwh
Output			
Bio-jet fuel	1	1	MJ
CO_2_ (biogenic)	86.7	163	g
CO_2_ (nonbiogenic)	44.0	9.29	g

#### Simulation 4: Lignin gasification with hog fuel (LG-HF bio-jet)

The LG-HF bio-jet process is similar to the LG bio-jet process. The majority of the hydrogen demand is met via poplar lignin gasification. Natural gas steam reforming supplies the remaining hydrogen. Hog fuel is used to provide heat and steam for the facility. Major process inputs and outputs for the LG-HF bio-jet simulation are listed in Table 5. When the total amount of biomass used to produce bio-jet fuel is factored into yield calculations (poplar + hog fuel), the LG-HF bio-jet fuel yield is 200 L t^−1^.

### Ancillary chemicals

Each biorefinery process requires chemical inputs to convert the poplar biomass to jet fuel. The production of these chemicals is grouped into the ancillary chemicals section. Tables 4 and 5 list the major chemical inputs required by each biorefinery. Unit process data for the chemical inputs come from the USLCI [27], EcoInvent [28], literature, and the private sector. The electricity source in each unit process was set to come from a unit process representative of the 2012 U.S. national grid [34]. Data for enzyme production is supplied from Novozymes for their Cellic Ctec3 cellulases (Novozymes, personal communication, 2012). Transportation distances for each chemical are determined using the 2007 U.S. commodity flow survey [35].

### Fuel distribution and use

The bio-jet fuel produced from each biorefinery is assumed to be transported 640 km to a distribution center (round trip). End use of the bio-jet fuel is combustion in a jet engine. The bio-jet fuel produced in the biorefineries is assumed to be identical to petroleum-based Jet-A fuel and will therefore have the same emissions when combusted in a jet engine. The Greenhouse Gases, Regulated Emissions, and Energy Use in Transportation (GREET) is used to provide jet engine emissions Model v1.2.0.11425 (GREET).

## Abbreviations

FFU: fossil fuel use
GHGs: greenhouse gases
GWP: global warming potential
HF: hog fuel
LCA: life cycle assessment
LG: lignin gasification
NGSR: natural gas steam reforming
